# Worsening epidemiological situation of carbapenemase-producing Enterobacteriaceae in Europe, assessment by national experts from 37 countries, July 2018

**DOI:** 10.2807/1560-7917.ES.2019.24.9.1900123

**Published:** 2019-02-28

**Authors:** Alma Brolund, Nina Lagerqvist, Sara Byfors, Marc J Struelens, Dominique L Monnet, Barbara Albiger, Anke Kohlenberg, European Antimicrobial Resistance Genes Surveillance Network (EURGen-Net) capacity survey group

**Affiliations:** 1 Public Health Agency of Sweden, Solna, Sweden; 2 These authors contributed equally to this work; 3 European Public Health Microbiology Training Programme (EUPHEM), European Centre for Disease Prevention and Control, Stockholm, Sweden; 4 European Centre for Disease Prevention and Control, Stockholm, Sweden; 5 The members of the capacity survey group are listed at the end of this article

**Keywords:** Escherichia coli, Klebsiella pneumoniae, carbapenem resistance, carbapenemase-production, national surveillance of resistance

## Abstract

A survey on the epidemiological situation, surveillance and containment activities for carbapenemase-producing Enterobacteriaceae (CPE) was conducted in European countries in 2018. All 37 participating countries reported CPE cases. Since 2015, the epidemiological stage of CPE expansion has increased in 11 countries. Reference laboratory capability, dedicated surveillance and a specific national containment plan are in existence in 33, 27 and 14 countries, respectively. Enhanced control efforts are needed for CPE containment in Europe.

The rapid worldwide dissemination of carbapenemase-producing Enterobacteriaceae (CPE) represents a global threat to patient safety and healthcare systems [1,2]. While data based on invasive isolates from the European Antimicrobial Resistance Surveillance Network (EARS-Net) show stable proportions of carbapenem resistance in *Klebsiella pneumoniae* and *Escherichia coli* for the last 4 years in the European Union (EU)/European Economic Area (EEA) as a whole, there is considerable heterogeneity across EU/EEA countries, with proportions of carbapenem resistance in *K. pneumoniae* invasive isolates ranging from 0 to 65% in 2017 [3]. Furthermore, there are recent reports of spread of CPE in individual European countries [4-7].

In 2017, the European Centre for Disease Prevention and Control (ECDC) established the European Antimicrobial Resistance Genes Surveillance Network (EURGen-Net) to perform structured surveys of carbapenem- and/or colistin-resistant Enterobacteriaceae (CCRE) in Europe, building on the ’European Survey of Carbapenemase-Producing Enterobacteriaceae (EuSCAPE)’ project [8-10]. This network continues to support laboratory capacity building for detection and surveillance of CCRE in Europe. To determine the current epidemiological situation of CPE and the national capacity for surveillance and containment of CPE and/or carbapenem-resistant Enterobacteriaceae (CRE), a questionnaire was sent to national experts from 30 EU/EEA and seven EU candidate or potential candidate countries in June 2018. Here, we present the results of the 2018 assessment and compare them with previous assessments using the same methodology.

## EURGen-Net capacity survey and epidemiological staging system

**Figure 1:**
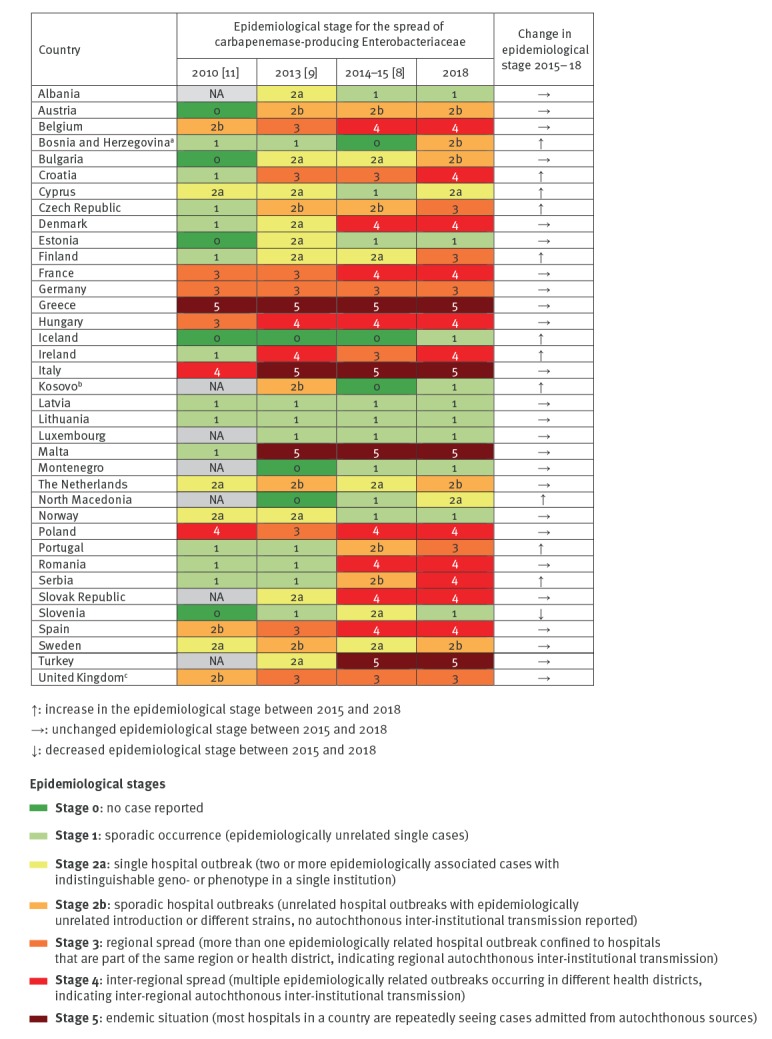
Comparison of epidemiological stages of carbapenemase-producing Enterobacteriaceae in European countries, 2010–2018 (n = 37)

The epidemiological staging system for measuring the extent of CPE spread across healthcare facilities was developed in 2010 and used in similar surveys in 2010, 2013 and 2015 [8,9,11]. The system consists of seven consecutive stages (0, 1, 2a, 2b, 3, 4 and 5) that describe national spread of CPE, from no cases (stage 0) to an endemic situation (stage 5) as reported in Figure 1. For EURGen-Net, the decision was made to extend the scope from CPE to CRE. Therefore, in this follow-up survey for the assessment of country capacity, both definitions were used as indicated in this manuscript. National experts representing all 37 invited European countries completed this 2018 capacity survey. Their answers were based on knowledge of national surveillance data and/or their professional experience at the National Reference/Expert laboratory, Public Health institute or Ministry of Health. For the UK, individual replies regarding capacity for surveillance and containment were provided for England, Northern Ireland, Wales and Scotland, and the corresponding information is reported separately where relevant.

NA: no data available.

^a^ The results reported for Bosnia and Herzegovina only apply to Republika Srpska.

^b^ This designation is without prejudice to positions on status, and is in line with United Nations Security Council Resolution 1244/99 and the International Court of Justice Opinion on the Kosovo declaration of independence.

^c^ The results reported are for the United Kingdom (UK) overall. The epidemiological stages vary between regions within the UK.

## Epidemiological situation of carbapenemase-producing Enterobacteriaceae

**Figure 2:**
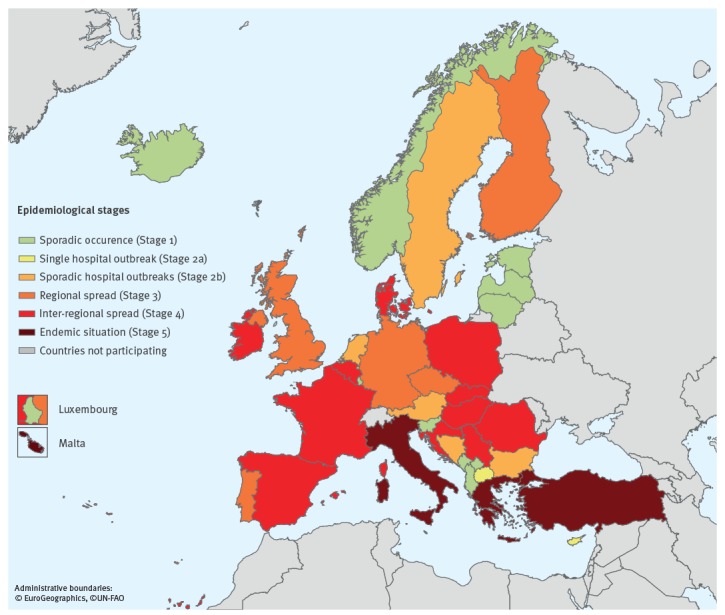
Epidemiological situation of carbapenemase-producing Enterobacteriaceae, assessment by national experts in European countries, July 2018 (n = 37)

In 2018, all 37 participating countries reported CPE cases, whereas in 2015, three countries had still not identified a single case [8]. Overall, 11 countries reported a higher epidemiological stage of CPE than in 2015, 25 countries described no change and one country reported, after control of a hospital outbreak [12], an improvement of the CPE epidemiological situation [8] (Figure 1). Compared with 2015, four additional countries reported regional or inter-regional spread in 2018, thus increasing the number of countries with regional or inter-regional spread to 16. The same four countries as in 2015 (Greece, Italy, Malta and Turkey) reported an endemic situation in 2018 (Figures 1 and 2).

## National surveillance and notification of cases

In July 2018, 27 countries had a dedicated national surveillance system for CRE and three countries reported that they were developing such a system (Table 1). Twenty-four countries had national recommendations or obligations for notification of CRE cases to health authorities (Table 1). Notification to health authorities was mandatory for all detected CRE cases (i.e. CRE infections and colonisations) in 18 countries. Reporting was mandatory for all laboratories in 13 countries. In Greece, Italy and Ireland, notification was mandatory for invasive infections only, and mandatory notification in Belgium was enacted only in outbreak situations.

**Table 1:** National capacity for surveillance and containment of carbapenem-resistant and carbapenemase-producing Enterobacteriaceae, European countries, July 2018 (n = 37)

Country		National reference or expert laboratory	National surveillance system for CRE	National recommendation or obligation for notification for CRE	National plan for containment of CPE	National recommendation or guideline on infection control measures for CPE
Albania		**Y**	N	N	N	N
Austria		**Y**	**Y^a^**	N^b^	N	N
Belgium		**Y**	**Y^a^**	**Y^c^**	**Y**	**Y**
Bosnia and Herzegovina^d^		N	N	**Y^e^**	N	U
Bulgaria		**Y**	IP (2018)^f^	IP (2018)	IP (2018)	IP (2018)
Croatia		**Y**	**Y^g^**	**Y^e,h^**	**Y**	**Y**
Cyprus		**Y**	**Y^g^**	N	N	**Y**
Czech Republic		**Y**	**Y^g^**	**Y^e,h^**	**Y**	**Y**
Denmark		**Y**	**Y^a^**	IP (2018)^i^	**Y**	IP (2018)
Estonia		N	IP (2020)	IP (2020)^j^	N	N
Finland		**Y^k^**	**Y^g^**	**Y^e,h^**	**Y**	**Y**
France		**Y**	**Y^a^**	**Y^e^**	**Y**	**Y**
Germany		**Y**	**Y^a^**	**Y^e, h^**	N	**Y**
Greece		**Y^k^**	**Y^g^**	**Y^h, l^**	**Y**	**Y**
Hungary		**Y**	**Y^a^**	**Y^e^**	IP (2019)	**Y**
Iceland		**Y**	**Y^g^**	**Y^e, h^**	**Y**	**Y**
Ireland		**Y**	**Y^a^**	**Y^m^**	**Y**	**Y**
Italy		**Y^k^**	**Y^a^**	**Y^l^**	**Y**	**Y**
Kosovo^n^		IP	**Y^a^**	IP	IP	IP
Latvia		**Y**	**Y^g^**	**Y^e^**	N	N
Lithuania		**Y**	**Y^g^**	**Y^e,h^**	N	N
Luxembourg		**Y^k^**	**Y^a^**	IP (2019)	U	**Y**
Malta		**Y^k^**	**Y^a^**	**Y^e^**	IP (2018)^o^	**Y**
Montenegro		N	N	**Y^e, h^**	N	N
The Netherlands		**Y**	**Y^a^**	**Y**	N	**Y**
North Macedonia		**Y^k^**	**Y^g^**	IP (2019)	IP (2019)	IP (2019)
Norway		**Y**	**Y^g^**	**Y^e,h^**	IP (2018/2019)	**Y**
Poland		**Y**	**Y^a^**	**Y^e,h^**	**Y**	**Y**
Portugal		**Y**	**Y^g^**	**Y^e,h^**	**Y**	**Y**
Romania		**Y^k^**	IP (2019)^p^	**Y^q^**	IP (2019)	**Y**
Serbia		**Y**	N	N	N	N
Slovakia		**Y**	**Y^g^**	**Y^e,h^**	U	**Y**
Slovenia		**Y^k^**	N	N	N	**Y**
Spain		**Y**	**Y^a^**	**Y^e,h^**	**Y**	**Y**
Sweden		**Y**	**Y^g^**	**Y^e,h^**	**Y**	**Y**
Turkey		**Y**	N	N	N	IP
UK	England	**Y**	**Y^a^**	**Y^a^**	**Y**	**Y**
	Northern Ireland	**Y**	**Y^a^**	N	N	**Y**
	Scotland	**Y**	**N**	N	**Y**	**Y**
	Wales	**Y**	**Y^g^**	**Y^g^**	**Y**	**Y**

CRE: carbapenem-resistant Enterobacteriaceae; CPE: carbapenemase-producing Enterobacteriaceae; IP: in preparation (if a date of planned implementation was provided by the country, the year is shown in brackets); N: not in place; U: unknown (i.e. information not available to the respondent); UK: United Kingdom; Y: in place.

^a^ Voluntary participation.

^b^ In Austria, it is recommended for laboratories to report all suspected CPE isolates to the national reference laboratory.

^c^ In Belgium, reporting is mandatory for outbreak situations only.

^d^ The results for reported Bosnia and Herzegovina only apply to Republika Srpska.

^e^ Mandatory notification for all detected cases.

^f^ Bulgaria has a national surveillance system for antimicrobial resistance including CPE, but is planning to implement a dedicated system for CRE.

^g^ Mandatory participation.

^h^ Mandatory notification for all laboratories.

^i^ In Denmark, CPE have been made reportable from 5 September 2018 onwards.

^j^ In Estonia, reporting of resistant microorganisms related to hospital-acquired infections is obligatory and implementation of specific recommendations for CRE is planned.

^k^ A national expert laboratory fulfils a similar role as a national reference laboratory.

^l^ Mandatory notification for specific infections only.

^m^ In Ireland, notification of invasive CPE isolates has been mandatory since 2013, with a national enhanced surveillance system for all new CPE isolates in place since 2016. Addition of mandatory notification to health authorities of all new CPE isolates is planned.

^n^ This designation is without prejudice to positions on status, and is in line with United Nations Security Council Resolution 1244/99 and the International Court of Justice Opinion on the Kosovo declaration of independence.

^o^ For Malta, the national containment plan had been completed at the time of publication.

^p^ Romania currently reports carbapenem-resistant isolates to the European Antimicrobial Resistance Surveillance Network (EARS-Net), but is planning for a dedicated national surveillance system for CRE.

^q^ In Romania, reporting of CPE is mandatory when found in hospitals.

## National capacity for infection control measures for carbapenemase-producing Enterobacteriaceae and national plan for containment

Twenty-four countries reported to have issued national recommendations or guidelines for infection control measures for patients with confirmed CPE and five countries reported preparing these recommendations (Table 1). In 2015, 23 countries had reported having infection control recommendations for CPE in place [8]. In 2018, a national plan for containment of CPE was available in 14 countries and was under preparation in another seven countries (Table 1). In 2015, only 10 countries had reported having a national containment plan for CPE [8].

## National laboratory capacity

Thirty-three countries reported having a NRL or an equivalent expert laboratory for CRE and one country was preparing for implementing a NRL at the time of the assessment (Table 1). Thirty-two of these NRLs or equivalent laboratories had the capacity to perform genotypic identification or characterisation of CPE (Table 2). PCR and real-time PCR were the most frequently used molecular methods (n = 21 and n = 16, respectively) (Table 2).

**Table 2:** Laboratory capacity for genotypic detection and characterisation of carbapenem-resistant Enterobacteriaceae at national reference and expert laboratories, European countries, July 2018 (n = 37)

Country	Genotypic characterisation at national reference or expert laboratory	Laboratory methods for genetic characterisation used at national reference laboratory or expert laboratory			
		PCR	Real-time PCR	Single-gene sequencing	WGS
Albania	N	NL	NL	NL	NL
Austria	**Y**	**Y**	N	N	N
Belgium	**Y**	**Y**	N	**Y**	**Y**
Bosnia and Herzegovina^a^	NL	NL	NL	NL	NL
Bulgaria	**Y**	**Y**	**Y**	**Y**	N
Croatia	**Y**	**Y**	**Y**	**Y**	N
Cyprus	**Y**	N	**Y**	N	N
Czech Republic	**Y**	**Y**	**Y**	**Y**	**Y**
Denmark	**Y**	N	N	N	**Y**
Estonia	NL	NL	NL	NL	NL
Finland	**Y**	N	**Y**	N	**Y**
France	**Y**	**Y**	N	**Y**	**Y**
Germany	**Y**	**Y**	N	**Y**	**Y**
Greece	**Y**	**Y**	N	**Y**	N
Hungary	**Y**	**Y**	N	**Y**	**Y**
Iceland	**Y**	N	**Y**	N	N
Ireland	**Y**	N	**Y**	N	**Y**
Italy	**Y**	**Y**	N	**Y**	**Y**
Kosovo^b^	NL	NL	NL	NL	NL
Latvia	**Y**	N	**Y**	N	N
Lithuania	**Y**	**Y**	N	N	N
Luxembourg	**Y**	N	**Y**	N	**Y**
Malta	**Y**	N	**Y**	N	N
Montenegro	NL	NL	NL	NL	NL
The Netherlands	**Y**	**Y**	N	N	**Y**
North Macedonia	**Y**	**Y**	**Y**	N	N
Norway	**Y**	N	**Y**	N	**Y**
Poland	**Y**	**Y**	N	**Y**	**Y**
Portugal	**Y**	**Y**	**Y**	**Y**	**Y**
Romania	**Y**	**Y**	N	**Y**	N
Serbia	**Y**	**Y**	N	N	N
Slovakia	**Y**	**Y**	N	N	N
Slovenia	**Y**	N	**Y**	**Y**	N^c^
Spain	**Y**	**Y**	**Y**	**Y**	**Y**
Sweden	**Y**	N	N	N	**Y**
Turkey	**Y**	**Y**	N	N	N
United Kingdom	**Y**	**Y**	**Y**	N	N

Y: used; N: not used; NL: no national reference or expert laboratory; WGS: whole genome sequencing.

^a^ The results for reported Bosnia and Herzegovina only apply to Republika Srpska.

^b^ This designation is without prejudice to positions on status, and is in line with United Nations Security Council Resolution 1244/99 and the International Court of Justice Opinion on the Kosovo declaration of independence.

^c^ WGS has become available in Slovenia at the time of publication.

Sixteen NRLs or equivalent laboratories reported performing genotypic characterisation of carbapenem resistance mechanisms with whole genome sequencing (WGS) and 14 reported using single-gene sequencing, either in addition to WGS (n = 9) or as the only sequencing method (n = 5). All laboratories performing genotypic characterisation could identify *bla*_VIM_, *bla*_KPC_, *bla*_NDM_ and *bla*_OXA-48 like_, i.e. the most common carbapenemase genes [13]. Twenty-six laboratories reported testing for additional carbapenemase genes such as *bla*_IMP_, *bla*_GES_, *bla*_FRI_, *bla*_IMI_ and *bla*_SME_ on a regular basis or when rare carbapenemases were suspected. Nine countries reported having national guidelines for clinical laboratories on molecular detection and/or characterisation of carbapenem resistance determinants in Enterobacteriaceae; however, further information on the implemented methods in individual clinical laboratories within countries was not collected within this survey. 

## Discussion

The results of this joint assessment by national experts of the epidemiological situation in 37 European countries indicates that CPE in healthcare systems in Europe disseminated further between 2015 and 2018. In 2018, 20 of 37 countries reported inter-institutional spread of CPE within the country (epidemiological stages 3–5) and 11 countries reported a worsened epidemiological situation compared with 2015 [8]. While this trend may in some countries be partially be explained by increased awareness and ascertainment capability through improved surveillance and diagnostic capacity, the latter was already largely in place in the majority of EU/EEA countries in 2015 at the end of the EuSCAPE project [14], as confirmed by the EULabCap 2016 survey [15]. However, it cannot be excluded that there is still underdetection of CPE in some countries depending on the frequency of microbiological sampling and of screening for CPE. Therefore, the direct comparison between countries with different levels of healthcare infrastructure and resources should be made with caution. In addition, the 2010 survey is only of limited use as a baseline as this survey was initiated very early in the upsurge of CPE and only minimal information was available in many countries at this time.

Advanced technical capacity for laboratory detection, molecular investigation and surveillance of CRE/CPE was widely available in the participating countries in 2018, especially in countries in the EU and EEA. The use of WGS for national surveillance and investigation of CRE/CPE has rapidly increased in EU/EEA countries since 2013 [16]. This was confirmed in our survey, with 16 EU/EEA countries reporting that they used this method for molecular investigation of CRE/CPE in 2018. Nevertheless, there are areas for improvement regarding prevention and control policies as more than half of the surveyed countries lacked national plans for containment of CPE and about a third did not have a recommendation or guideline on infection control measures for CPE. With many occurrences of cross-border transfer of CPE reported globally and within Europe [17-19], the success of CPE control activities depends on all countries having strong surveillance and control measures in place.

These results do not provide information on the factors driving the apparent increasing dissemination of CPE in Europe, which would require further investigations. In this context, a structured survey of CCRE isolates is planned within EURGen-Net and approximately 300 European hospitals will collect CCRE isolates with related epidemiological information in 2019 [20]. The isolates will be analysed by WGS to determine the presence and distribution of high-risk CCRE clones, epidemic clades and successful plasmids carrying carbapenemase genes in European hospitals and their potential cross-border spread [20]. A more in-depth understanding of the molecular epidemiology and transmission routes of CPE/CRE in Europe can inform risk assessment and allow better targeting of prevention and control efforts.

## Conclusion

The presented results show that the ongoing dissemination of CPE is further expanding across healthcare systems in Europe. This trend highlights the need for enhanced containment efforts within countries as well as concerted action at a European level.

## EURGen-Net capacity survey group

Austria: Petra Apfalter, Rainer Hartl. Belgium: Youri Glupczynski, Daniel Te-Din Huang. Bulgaria: Ivan Ivanov, Stefana Sabtcheva. Croatia: Irina Pristas, Arjana Tambić Andrašević. Cyprus: Panagiota Maikanti-Charalampous. Czech Republic: Jaroslav Hrabak. Denmark: Anette M. Hammerum, Ute W. Sönksen. Estonia: Anastasia Bilozor, Marina Ivanova. Finland: Outi Lyytikäinen, Kati Räisänen. France: Mélanie Colomb-Colinat, Laurent Dortet. Germany: Ines Noll, Niels Pfennigwerth. Greece: Alkiviadis Vatopoulos. Hungary: Ákos Tóth. Iceland: Kristján Orri Helgason. Ireland: Wendy Brennan, Karen Burns. Italy: Monica Monaco, Annalisa Pantosti  Latvia: Oksana Savicka, Solvita Selderina. Lithuania: Aistė Mierauskaitė. Luxembourg: Monique Perrin. Malta: Nina Nestorova. Netherlands: Leo Schouls, Kim van der Zwaluw. Norway: Ørjan Samuelsen. Poland: Elżbieta Literacka, Dorota Żabicka. Portugal: Manuela Caniça, Vera Manageiro. Romania: Irina Codita, Gabriel Adrian Popescu. Slovakia: Milan Nikš, Andrea Žáková. Slovenia: Mateja Pirš, Maja Šubelj. Spain: José Campos, Jesús Oteo. Sweden: Petra Edquist, Karin Sjöström. United Kingdom: Katie Hopkins. Albania: Artan Bego, Andi Koraqi. Bosnia and Herzegovina: Maja Travar. Kosovo^a^: Arsim Kurti, Lul Raka. Montenegro: Milena Lopičić, Vineta Vuksanović. North Macedonia: Ana Kaftandziewa, Dugagjin Osmani. Serbia: Ivana Cirkovic, Deana Medić. Turkey: Hüsniye Şimşek, Serap Süzük Yildiz.

^a^ This designation is without prejudice to positions on status, and is in line with United Nations Security Council Resolution 1244/99 and the International Court of Justice Opinion on the Kosovo declaration of independence.

## References

[1] European Centre for Disease Prevention and Control (ECDC). Carbapenem-resistant Enterobacteriacae, first update. Rapid risk assessment. Stockholm: ECDC; 2018. Available from: https://ecdc.europa.eu/sites/portal/files/documents/RRA-Enterobacteriaceae-Carbapenems-European-Union-countries.pdf

[2] Logan LK Weinstein RA The epidemiology of carbapenem-resistant Enterobacteriaceae: The impact and evolution of a global menace. J Infect Dis. 2017;215(suppl_1):S28-36. 10.1093/infdis/jiw282 28375512 PMC5853342

[3] European Centre for Disease Prevention and Control (ECDC). Surveillance of antimicrobial resistance in Europe. Annual report of the European Antimicrobial Resistance Surveillance Network (EARS-Net) 2017. Stockholm: ECDC; 2018. Available from: https://ecdc.europa.eu/sites/portal/files/documents/AMR-surveillance-EARS-Net-2017.pdf

[4] Guducuoglu H Gursoy NC Yakupogullari Y Parlak M Karasin G Sunnetcioglu M Hospital outbreak of a colistin-resistant, NDM-1- and OXA-48-producing Klebsiella pneumoniae: high mortality from pandrug resistance. Microb Drug Resist. 2018;24(7):966-72. 10.1089/mdr.2017.0173 29265963

[5] Avgoulea K Di Pilato V Zarkotou O Sennati S Politi L Cannatelli A Characterization of extensively drug-resistant or pandrug-resistant sequence type 147 and 101 OXA-48-producing Klebsiella pneumoniae causing bloodstream infections in patients in an intensive care unit. Antimicrob Agents Chemother. 2018;62(7):e02457-17. 10.1128/AAC.02457-17 29661874 PMC6021667

[6] Brkic DV Pristas I Cipris I Jelic M Butic I Andrasevic AT Successful containment of the first KPC-producing Klebsiella pneumoniae outbreak in Croatia. Future Microbiol. 2017;12(11):967-74. 10.2217/fmb-2016-0143 28795847

[7] Kaiser T Finstermeier K Häntzsch M Faucheux S Kaase M Eckmanns T Stalking a lethal superbug by whole-genome sequencing and phylogenetics: Influence on unraveling a major hospital outbreak of carbapenem-resistant Klebsiella pneumoniae. Am J Infect Control. 2018;46(1):54-9. 10.1016/j.ajic.2017.07.022 28935481

[8] Albiger B Glasner C Struelens MJ Grundmann H Monnet DL European Survey of Carbapenemase-Producing Enterobacteriaceae (EuSCAPE) working group Carbapenemase-producing Enterobacteriaceae in Europe: assessment by national experts from 38 countries, May 2015. Euro Surveill. 2015;20(45):30062. 10.2807/1560-7917.ES.2015.20.45.30062 26675038

[9] Glasner C Albiger B Buist G Tambić Andrasević A Canton R Carmeli Y Carbapenemase-producing Enterobacteriaceae in Europe: a survey among national experts from 39 countries, February 2013. Euro Surveill. 2013;18(28):20525. 10.2807/1560-7917.ES2013.18.28.20525 23870096

[10] European Centre for Disease Prevention and Control (ECDC). Carbapenemase-producing bacteria in Europe. Interim results from the European survey on carbapenemase-producing Enterobacteriaceae (EuSCAPE) project 2013. Stockholm: ECDC; 2013. Available from: https://ecdc.europa.eu/sites/portal/files/media/en/publications/Publications/antimicrobial-resistance-carbapenemase-producing-bacteria-europe.pdf

[11] Grundmann H Livermore DM Giske CG Canton R Rossolini GM Campos J Carbapenem-non-susceptible Enterobacteriaceae in Europe: conclusions from a meeting of national experts. Euro Surveill. 2010;15(46):19711. 10.2807/ese.15.46.19711-en 21144429

[12] Pirš M Cerar Kišek T Križan Hergouth V Seme K Mueller Premru M Jeverica S Successful control of the first OXA-48 and/or NDM carbapenemase-producing Klebsiella pneumoniae outbreak in Slovenia 2014-2016. J Hosp Infect. 2019;101(2):142-9. 10.1016/j.jhin.2018.10.022 30399389

[13] Meletis G Carbapenem resistance: overview of the problem and future perspectives. Ther Adv Infect Dis. 2016;3(1):15-21. 10.1177/2049936115621709 26862399 PMC4735501

[14] Grundmann H Glasner C Albiger B Aanensen DM Tomlinson CT Andrasević AT Occurrence of carbapenemase-producing Klebsiella pneumoniae and Escherichia coli in the European survey of carbapenemase-producing Enterobacteriaceae (EuSCAPE): a prospective, multinational study. Lancet Infect Dis. 2017;17(2):153-63. 10.1016/S1473-3099(16)30257-2 27866944

[15] European Centre for Disease Prevention and Control (ECDC). EU Laboratory Capability Monitoring System (EULabCap): Report on 2016 survey of EU/EEA country capabilities and capacities. Stockholm: ECDC; 2018. Available from: https://ecdc.europa.eu/en/publications-data/eu-laboratory-capability-monitoring-system-eulabcap-report-2016-survey-eueea

[16] European Centre for Disease Prevention and Control (ECDC). Monitoring the use of whole-genome sequencing in infectious disease surveillance in Europe 2015–2017. Stockholm: ECDC; 2018. Available from: https://www.ecdc.europa.eu/sites/portal/files/documents/whole-genome-sequencing-infectious-disease-surveillance-Europe-2015-2017.pdf

[17] European Centre for Disease Prevention and Control (ECDC). Carbapenemase-producing (OXA-48) Klebsiella pneumoniae ST392 in travellers previously hospitalised in Gran Canaria, Spain. Rapid risk assessment. Stockholm: ECDC; 2018. Available from: https://ecdc.europa.eu/sites/portal/files/documents/28-06-2018-RRA-Klebsiella-pneumoniae-Spain-Sweden-Finland-Norway.pdf

[18] Hrabák J Papagiannitsis CC Študentová V Jakubu V Fridrichová M Zemlickova H Carbapenemase-producing Klebsiella pneumoniae in the Czech Republic in 2011. Euro Surveill. 2013;18(45):20626. 10.2807/1560-7917.ES2013.18.45.20626 24229789

[19] Samuelsen Ø Overballe-Petersen S Bjørnholt JV Brisse S Doumith M Woodford N Norwegian Study Group on CPE Molecular and epidemiological characterization of carbapenemase-producing Enterobacteriaceae in Norway, 2007 to 2014. PLoS One. 2017;12(11):e0187832. 10.1371/journal.pone.0187832 29141051 PMC5687771

[20] European Centre for Disease Prevention and Control (ECDC). ECDC study protocol for genomic-based surveillance of carbapenem-resistant and/or colistin-resistant Enterobacteriaceae at the EU level. Version 2.0. Stockholm: ECDC; 2018. Available from: https://ecdc.europa.eu/en/publications-data/ecdc-study-protocol-genomic-based-surveillance-carbapenem-resistant-andor

